# Potential protective role of interferon-induced protein with tetratricopeptide repeats 3 (IFIT3) in COVID-19

**DOI:** 10.3389/fcimb.2024.1464581

**Published:** 2024-11-27

**Authors:** Mateus V. de Castro, Leonardo M. Cariste, Rafael R. Almeida, Greyce L. Sasahara, Monize V. R. Silva, Flávia B. Soares, Vivian R. Coria, Michel S. Naslavsky, Keity S. Santos, Edecio Cunha-Neto, Jorge Kalil, Mayana Zatz

**Affiliations:** ^1^ Human Genome and Stem Cell Research Center, University of São Paulo, São Paulo, SP, Brazil; ^2^ Laboratory of Immunology, Heart Institute (InCor), Hospital das Clínicas da Faculdade de Medicina da Universidade de São Paulo, (HCFMUSP), São Paulo, Brazil; ^3^ Instutute on Investigation in Immunology, - Instituto Nacional de Ciências e Tecnologia-iii-INCT, São Paulo, Brazil; ^4^ Division of Clinical Immunology and Allergy, Departamento de Clínica Médica, Faculdade de Medicina da Universidade de São Paulo, São Paulo, Brazil

**Keywords:** IFIT3, COVID-19, innate immunity, SARS-CoV-2, protection

## Abstract

The COVID-19 pandemic has prompted a quest to understand why certain individuals remain uninfected or asymptomatic despite repetitive exposure to SARS-CoV-2. Here, we focused on six exposed females residing with their symptomatic and reinfected SARS-CoV-2 PCR-positive COVID-19 partners. Peripheral blood mononuclear cell samples from couples were analysed for poly (I:C)-induced mRNA expression of type I/III interferons and interferon-stimulated genes (ISGs). Remarkably, we found a significant upregulation of the ISG interferon-inducible protein with tetrapeptide repeats 3 (IFIT3) gene exclusively in exposed uninfected or asymptomatic females, suggesting a potential role in protective immunity against symptomatic COVID-19.

## Introduction

Natural resistance against respiratory (Trammell and Toth, 2008) and non-respiratory (Cook, 2015) viral infections has been previously suggested. Worldwide reports of individuals exposed to SARS-CoV-2 who remained uninfected support the existence of biological protective factors against SARS-CoV-2 infection (Andreakos et al., 2022; Netea et al., 2022; Zhang et al., 2022). Identifying protective mechanisms against SARS-CoV-2 infection can potentially contribute to new diagnostic and therapeutic strategies (Netea et al., 2022; Zhang et al., 2022). In a previous study of COVID-19 and SARS-CoV-2 PCR discordant couples with prolonged close contact, we have found that males are significantly more affected than female partners (Silva et al., 2022). This has been attributed mainly to the development of more finely tuned and effective immune responses against infections in females (Foresta et al., 2021). The Interferon type I and III response plays a major role in innate immunity against viral infection in general and in particular against SARS-CoV-2 (Watson et al., 2020). We here aimed to identify an innate immunity gene signature that could indicate why these females remained uninfected or asymptomatic despite being continuously exposed to their partners’ infections. We investigated poly (I:C)-induced expression of type I/III IFNs and IFN-stimulated genes (ISG) in peripheral blood mononuclear cells of SARS-CoV-2 serum-discordant couples. We selected to study ISG that acted at different levels in the type I/III IFN response to viral infection: IFIT3, IFITM1 and IRF7. Our results suggest a potential emerging role of the ISG interferon-inducible protein with tetrapeptide repeats 3 (IFIT3) gene in the protective immunity against symptomatic COVID-19.

The Interferon-Induced Proteins with Tetratricopeptide Repeats (IFIT) family plays a crucial role in the innate immune response, particularly in antiviral defense mechanisms (Zhang et al., 2023b; Chen et al., 2024; Wang et al., 2024). These genes are rapidly induced by type I and III interferons during viral infections, orchestrating the host’s immune defense (Burchill et al., 2021; Çağlayan and Kadir, 2022; Zhang et al., 2023a). The IFIT gene family includes several members, including IFIT1, IFIT2, IFIT3, and IFIT5, all of which share common structural feature—tetratricopeptide repeat (TPR) domains—that facilitate protein-protein interactions. These proteins contribute to the immune response by inhibiting viral replication, interfering with viral RNA translation, and modulating the antiviral effects of interferons (Xu et al., 2022). Among the family members, IFIT3 has garnered significant attention for its emerging role in host defense against a wide range of viral pathogens (Wu et al., 2022; Franco et al., 2023). Studies have demonstrated that IFIT3 not only works in concert with other IFIT family members but also functions independently to restrict viral spread. It achieves this by binding to viral RNA, inhibiting translation, and regulating the antiviral state of the cells (Sfikakis et al., 2021; Xu et al., 2022). Additionally, IFIT3 plays a critical role in amplifying the interferon response by stabilizing IFIT1 and IFIT2 complexes, further enhancing their antiviral effects; in the context of HBV infection, IFIT3 overexpression upregulated ISGs such as MxA, OAS1 and PKR, in a STAT2-dependent manner (Chai et al., 2021; Chikhalya et al., 2021; Imaizumi et al., 2021; Xu et al., 2022). Given these properties, the IFIT gene Family - particularly IFIT3 - represents a promising area of study for understanding the protective mechanisms in individuals who remain uninfected or asymptomatic despite exposure to SARS-CoV-2.

## Methods

### Volunteers’ recruitment, blood collection, and sample processing

The six couples evaluated here belong to a larger cohort named “Brazilian Discordant Couples” ascertained at the Human Genome and Stem Cell Research Center - HUG-CELL (Castelli et al., 2021). In the previous phase of our study, conducted by the end of 2020, we recruited 100 couples from Sao Paulo City (the largest city in Brazil). In these pairs, one member had symptomatic COVID-19 and had a SARS-CoV-2 PCR-positive oropharyngeal swab, while the partner remained uninfected (as shown by a negative result in oropharyngeal swab SARS-CoV-2 PCR) despite sharing the same bed throughout the infection. Blood collection occurred at least one month after the initial illness in the affected partner, before COVID-19 vaccination in Brazil, and before the emergence of new SARS-CoV-2 variants. We conducted serological testing to confirm SARS- CoV-2 infection status and serum discordance. Interestingly, some “uninfected” individuals displayed IgG antibodies, and these couples were excluded from further analysis. The study proceeded with 83 serum-discordant couples we followed annually. By the end of 2022, only 36 individuals (from COVID-19 seronegative group) remained uninfected or had a subclinical infection (asymptomatic volunteers). As the individuals in the study cohort received vaccinations, serological tests would detect the presence of antibodies, making it impossible to distinguish between antibodies resulting from natural infection or vaccine-induced immunity. Notably, among these 36 cases, there were six instances where the male partner had been reinfected (confirmed by a positive oropharyngeal swab SARS-CoV-2 PCR test) and the female partner remained uninfected or asymptomatic. It is also important to highlight that these females did not exhibit any flu-like illness or COVID-19-related symptoms at any point during the pandemic.

Peripheral blood samples of these six couples were then collected in vacutainer tubes with sodium heparin (BD Biosciences, reference #367874) to obtain peripheral blood mononuclear cells (PBMCs) after Ficoll density gradient centrifugation. Five other age-matched females with symptomatic COVID-19 before vaccination made up a control group. Mean age ± standard deviation: Reinfected male partners: 46 ± 5 years old; Exposed female partners: 47 ± 6 years old; Symptomatic females (Control): 48 ± 4 years old.

### Type I/III IFN innate immune response

To investigate type I, type III interferon responses and interferon-stimulated genes, we transfected PBMCs (1x10^6^ cells/well) with 200 ng/ul of the well-known type I and III IFN inducer Poly I:C (Invitrogen, USA) mixed with lipofectamine (Invitrogen, reference #11668019) in a final volume of 200 µl of Opti-MEM medium (Invitrogen, reference #31985062), according to the manufacturer’s instructions. The cells were incubated for 1, 4, and 8 hours at 37°C and 5% CO2 in 96-well plates. Negative controls were incubated in Opti-MEM medium and lipofectamine. Total RNA was obtained using the RNeasy Mini kit (Qiagen, Germany); after extraction, we treated RNA with a DNA removal kit (Invitrogen, reference #AM1906) according to the manufacturer’s instructions. The preparation of cDNA with Superscript II Reverse Transcriptase (Thermofisher Fisher Scientific, USA) was conducted according to the manufacturer’s instructions. mRNA levels for IFN-alpha (*IFNA2* gene), IFN-beta (*IFNB1* gene), IFN-lambda (*IFNL2* and *IFNL3* genes), IFN-stimulated genes (ISGs) as IFN regulatory factor 7 (*IRF7*), IFN induced protein with tetratricopeptide repeats 3 (*IFIT3*) and IFN Induced Transmembrane Protein 1 (*IFITM1*) were evaluated by real-time qPCR with Power SYBR Green Master Mix (Thermo Fisher Scientific, USA) on a QuantStudio 12 K flex (Applied Biosystems, USA). GAPDH was used as an endogenous control. Primers for the RT-PCR were designed using Primer-BLAST. **Table 1** presents the primers list. Graphics and statistical analyses were performed using GraphPad v.9.02 (Prism Software). Comparison of continuous variables was carried out using Kruskal– Wallis test with Dunn’s *post-hoc* test for several groups or Mann–Whitney test when only two groups were compared, with a significance level set at p < 0.05.

**Table 1:** Primers used for reverse transcription polymerase chain reaction (RT-PCR) to assess the innate type I/III IFN immune response.

Gene	Forward 5′ – 3’	Reverse 5′ – 3’
*IFNA2*	TCGTATGCCAGCTCACCTTT	TCGTGTCATGGTCATAGCAGAA
*IFNB1*	ACGCCGCATTGACCATCTAT	GTCTCATTCCAGCCAGTGCT
*IRF7*	CTTCGTGATGCTGCGGGATA	TTCTCGCCAGCACAGCTC
*IFIT3*	AAGAACAAATCAGCCTGGTCAC	GACCTCACTCATGACTGCCC
*IFITM1*	GCCAAGTGCCTGAACATCTG	TGTCACAGAGCCGAATACCAG
*IFNL2*	TCCCAGACAGAGCTCAAAACT	CAGTCCCCTCTTCTGGATCTC
*IFNL3*	ACGCGAGACCTGAATTGTGT	TCAGGTTGCATGACTGGCG
*GAPDH*	CTCTGCTCCTCCTGTTCGAC	ATGGTGTCTGAGCGATGTGG

### Single-cell RNA sequencing data analysis

To investigate the specific cell populations expressing the genes of interest, we explored publicly available single-cell RNA sequencing (scRNA-seq) data from COVID-19 patients. These data were obtained from online repositories. Our focus on whole PBMCs aimed to determine exactly which cells are expressing, or not, the evaluated genes (IFNA2, IFNB1, IRF7, IFIT3, IFITM1, IFNL2, and IFNL3). This approach allowed us to compare gene expression between COVID-19 patients and healthy controls, providing insights into the transcriptional landscape of PBMCs during infection (Lee et al., 2020). In addition, we investigated the expression of the interferon-related genes of interest in neutrophils, which are abundant in the airways of SARS-CoV-2 patients and express several genes associated with interferon signaling (Ren et al., 2021).

### Whole-exome sequencing

Whole-exome sequencing (WES) was conducted to analyze 31 interferon-stimulated genes (ISGs) involved in key stages of the antiviral response, including viral entry (e.g., IFITMs and CH25H), replication (e.g., Viperin), mRNA synthesis, protein synthesis, and viral egress, as described by Schoggins, 2021. The aim was to identify genetic variants that might influence these crucial immune processes and affect the host’s capacity to combat SARS-CoV-2 infection. Additionally, the analysis sought to detect any inborn errors of immunity (IEI) that could specifically impact the type I interferon (IFN-I) pathway, given their association with increased susceptibility to SARS-CoV-2 infection (Zhang et al., 2022). WES was performed on peripheral blood DNA using the Illumina NovaSeq platform at HUG-CELL facilities. Sequencing data were processed with the BWA-MEM and GATK Best Practices workflow, ensuring stringent quality control and comprehensive annotation, following previously described protocols (Castelli et al., 2021).

## Results

**Figure 1:**
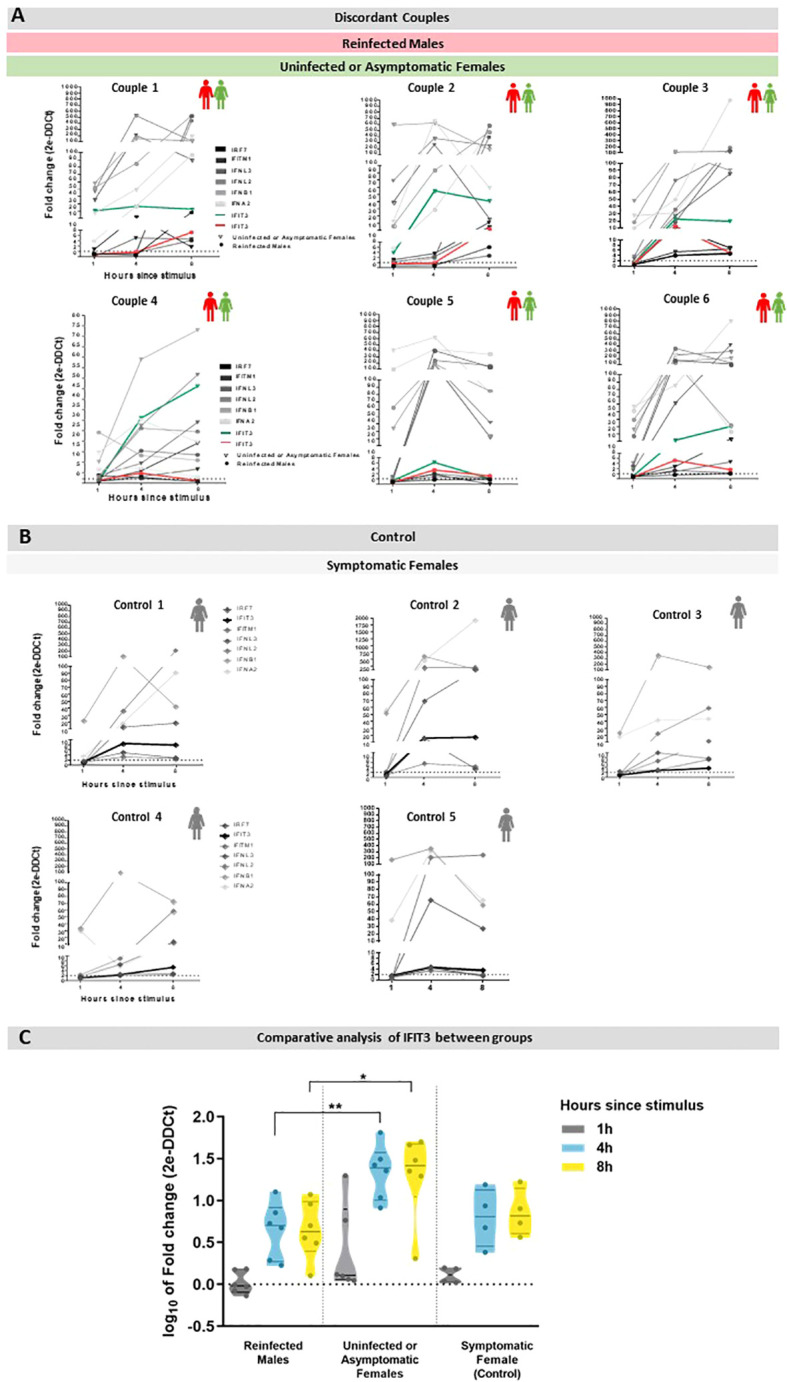
Expression of type I/III IFNs and ISGs in PBMC transfected with Poly (I:C) for 1, 4, and 18h. Data are presented as gene expression in the stimulated cells relative to unstimulated cells. **(A)** Gene expression in discordant couples, in which the green lines represent IFIT3 expression in uninfected or asymptomatic females for COVID-19, and red lines represent IFIT3 expression in COVID-19 reinfected males. **(B)** Gene expression in symptomatic females for COVID-19 (control), in which bold lines represent the expression of IFIT3. **(C)** Comparative analysis of different IFIT3 gene expressions between reinfected males, uninfected or asymptomatic females, and symptomatic females after stimulus; the data represent a statistically significant increase at 4 and 8h between the uninfected or asymptomatic females and reinfected males group, results are represented by mean ± SD. **p < 0.009 *p < 0.05.

After transfection with poly (I:C), PBMC’s from all subjects exhibited an early and robust (fold change = 20 or higher) mRNA expression of at least one of the four type I/III interferon (IFN) genes assayed (IFN-I, represented by IFNA2 (IFN-α) and IFNB1 (IFN-β), and IFN-III, represented by IFNL2 and IFNL3 (IFN-λ), at the earliest time point (1h) following stimulation (**Figure 1**).

Interestingly, a significant upregulation in the expression of the interferon-induced gene *IFIT3* was observed 4h after poly (I:C) stimulation only in the females who were exposed to their reinfected male partners but remained uninfected or were asymptomatic. Notably, the elevated expression of *IFIT3* persisted 8h after the stimulus in these individuals. Importantly, the observation that the infected females (control group) exhibited a gene expression profile similar to the reinfected males ruled out a sex-specific phenotype. *IFIT3* is the only gene with noteworthy differences between reinfected male and uninfected/asymptomatic female subjects.

Baseline expression data for each time point (1h, 4h, and 8h) for interferon-stimulated genes (ISGs) in the three groups are presented in **Supplementary Figure 1**. Upon analysis, no statistically significant differences were observed among the groups. This information suggests that the differences in *IFIT3* gene expression fold change following *in vitro* stimulation cannot be attributed to baseline variations between the groups.

To enhance the analysis and provide a deeper understanding of the cellular context of the evaluated genes, we compared our dataset with publicly available single-cell RNA sequencing (scRNA-seq) datasets of PBMCs from COVID-19 patients, as well as evaluated the expression in neutrophils, which are the most abundant cell type in the airways of SARS-CoV-2 patients. By integrating our data with these scRNA-seq datasets, we identified specific immune cell populations that exhibited upregulation of key interferon-stimulated genes (ISGs), including IFIT3 (**Supplementary Figure 2**). Notably, IFIT3 was found to be broadly expressed across multiple immune cell types, suggesting its significant role in antiviral responses, particularly within monocytes and antigen-presenting cells (**Supplementary Figure 2A**). Additionally, elevated expression levels of IFIT3 and other ISGs, such as IFITM1, were observed in neutrophils from COVID-19 patients (**Supplementary Figure 2B**). This increased expression in neutrophils underscores their involvement in the immune defense against SARS-CoV-2, contributing to a more effective response in controlling viral propagation. These findings emphasize the importance of cell-specific expression patterns in driving the overall antiviral defense, further validating the relevance of our gene expression data.

Finally, we have also searched in our cohort for potentially high impact pathogenic variants across a list of genes associated with IFN. Besides common variants (gnomAD v4.1 frequency > 1%), no rare variants with deleterious consequences were found except for a stop gain alteration in IFNL2 gene (NM_172138.2):c.562C>T:p.(Arg188Ter), rs200581777. The variant is rare worldwide, reaching a maximum allelic frequency of 0.4% in Middle Eastern cohorts of gnomAD. This variant was found in heterozygosity in one subject (the reinfected male partner from couple 3). Even though a potential loss of function in a single allele is not likely to cause viral infection susceptibility, its influence as a risk factor may not be excluded.

## Discussion

Our main finding reveals an overexpression of the interferon-inducible protein with tetrapeptide repeats 3 (IFIT3) gene exclusively in uninfected or asymptomatic females who were repeatedly exposed to their symptomatic COVID-19 male partners, suggesting a potential protective role of IFIT3 expression against development of symptomatic COVID-19. On the other hand, induction of type I/III IFN was shared among all groups.

While a previous study failed to associate plasma IFN-α levels to SARS-CoV-2 exposed uninfected individuals (Norton et al., 2023) no previous studies analyzed the induction of IFNs or Interferon-stimulated genes in PBMC from exposed uninfected and/or asymptomatic patients. Many RNA and DNA viruses are known to induce the expression of IFIT3, thereby inhibiting viral replication by regulating immune-related molecules or pathways within the antiviral innate immunity framework (Zhang et al., 2023b). In response to *in vitro* SARS-CoV-2 infection, human airway epithelium demonstrates an increased level of IFIT3 mRNA (Assou et al., 2023). The overexpression of IFIT3 was shown to efficiently suppress the replication of RNA virus swine influenza virus (SIV), whereas knockdown of IFIT3 increases SIV replication (Li et al., 2015). Functionally, the human IFIT3 protein stimulates interferon (IFN) production through mitochondrial antiviral signaling (MAVS) while inhibiting adenovirus immediate early gene expression (Chikhalya et al., 2021). Further, a CRISPR-based loss-of-function screen identified IFIT3 as a pivotal interferon-stimulated gene in the interferon-mediated restriction of RNA virus Venezuelan equine encephalitis virus (McDougal et al., 2023).

Interestingly, IFIT3 has been found to enhance the antiviral effect of IFN-α through the JAK-STAT pathway, concurrently impeding cell proliferation in hepatitis B virus infection (Xu et al., 2022). Additionally, suggestive evidence points towards the antiviral role of IFIT3 in dengue virus infection, as a deficiency in IFIT3 is associated with increased viral production in human lung epithelial cells (Hsu et al., 2013). Indeed, it was found that the expression of a single ISG, IFIT3, establishes a cellular antiviral state independent of viral PAMPs (Chikhalya et al., 2021). These findings imply a crucial role for IFIT3 in innate immunity against viruses, specifically contributing to the protection of symptomatic COVID-19.

The scRNA-seq data further reinforce the importance of understanding cell-specific responses by showing distinct expression patterns of interferon-stimulated genes (ISGs) across different immune cell types in PBMCs from COVID-19 patients. Notably, IFIT3 was broadly expressed across various immune cells, including monocytes and antigen-presenting cells, highlighting its significant role in antiviral defense. Additionally, the elevated expression of IFIT3 and other ISGs, such as IFITM1, was observed in neutrophils, which are the most abundant cell type in the airways of SARS-CoV-2 patients. Traditionally considered to play a limited role in interferon signaling, neutrophils exhibited a unique transcriptional profile during SARS-CoV-2 infection, suggesting they contribute more significantly to the immune response in COVID-19 than previously recognized. Interestingly, low-density neutrophils - a specific population of neutrophils - are also found in PBMC in addition to monocytes, and low density neutrophil-associated proteins were found to be increased in proteomic profiling of PBMC from COVID-19 patients (Leite et al., 2023) suggesting this cell population might be the source of IFIT3 expression in PBMC. Of particular interest is the elevated expression of IFIT3, a gene with a well-established role in airway neutrophils and known for its protective function against SARS-CoV-2 infection, as demonstrated in a previous study (Margaroli et al., 2023). These findings align with recent evidence indicating that neutrophils are crucial in mounting an effective immune response to viral infections, especially in the context of COVID-19 (Xu et al., 2022).

One possible explanation for IFIT3 overexpression in certain individuals could be the presence of genetic variations or epigenetic modifications that enhance IFIT3 expression in response to viral exposure without causing symptomatic infection. Additionally, environmental factors, such as repeated low-dose viral exposure from cohabiting with infected partners, might prime the immune system, leading to a heightened state of antiviral readiness. This heightened response could be more pronounced in females due to hormonal influences, particularly the immunomodulatory effects of estrogen, which has been shown to enhance the expression of interferon-stimulated genes. Moreover, differences in immune system regulation between sexes, such as variations in the expression of toll-like receptors and interferon signaling pathways, may also contribute to the observed disparity. The mechanisms whereby some individuals overexpress IFIT3 after induction and are protected from SARS-CoV-2 infection remain to be investigated, but their elucidation may lead to potential therapeutic targets.

While our study focused on gene expression analysis through quantitative PCR, particularly after Poly (I) stimulation, we acknowledge the potential for a broader understanding through the application of next-generation sequencing (NGS) techniques. The observed differential regulation of IFIT3 led us to concentrate on this specific gene. However, RNA-seq or other NGS methods could provide a more comprehensive view of interferon-stimulated genes (ISGs) and identify additional candidates, such as IFITM, Viperin, and CH25H, that are likely involved in the antiviral response (Lee et al., 2020). By expanding on these findings, future research could delve deeper into the intricate immune response mechanisms that contribute to protection against symptomatic COVID-19.

### Study limitations

Despite the relatively low sample size, representing a small number of participants, these subjects were meticulously selected from a detailed subset of a cohort of “serum-discordant” couples for COVID-19. The cohort was diligently followed over three years, providing a nuanced and thorough examination of the selected cases. While the sample size may be limited, the depth of the cohort’s longitudinal follow-up enhances the granularity and context of the findings. In addition, it is essential to acknowledge that vaccination efforts were initiated during this period. As a result, it became challenging to distinguish between the immune response triggered by vaccination and that induced by natural infection. As the scientific literature suggests a protective role of the IFIT3 gene against other viral infections, it is highly likely that IFIT3 plays a significant role in COVID-19, as indicated by this study.

## Data Availability

The original contributions presented in the study are included in the article/**Supplementary Material**. Further inquiries can be directed to the corresponding author.

## Supplementary material

Supplementary Figure 2 The expression profiles of interferon-related genes (IFNA2, IFNB1, IRF7, IFIT3, IFITM1, IFNL2, and IFNL3) across specific immune cell types in COVID-19 patients, based on publicly available single-cell RNA sequencing (scRNA-seq) data. (A) Gene expression across various PBMC subtypes in blood samples from COVID-19 patients. Dot color represents gene expression levels, with a gradient from low (light yellow) to high (dark purple), while dot size indicates the percentage of cells within each population expressing each gene. (B) The expression levels of the same interferon-related genes in neutrophils from both normal (healthy) and COVID-19 conditions. Neutrophils, which are abundant in the airways of COVID-19 patients, play a key role in the antiviral immune response. The color gradient similarly represents gene expression intensity, and the dot size reflects the percentage of neutrophils expressing each gene in both conditions.
