# Supplementary figures and images for: Potential protective role of interferon-induced protein with tetratricopeptide repeats 3 (IFIT3) in COVID-19
Source: Front Cell Infect Microbiol. 2024 Nov 27;14:1464581. doi: 10.3389/fcimb.2024.1464581 (PMC11631949; doi:10.3389/fcimb.2024.1464581)

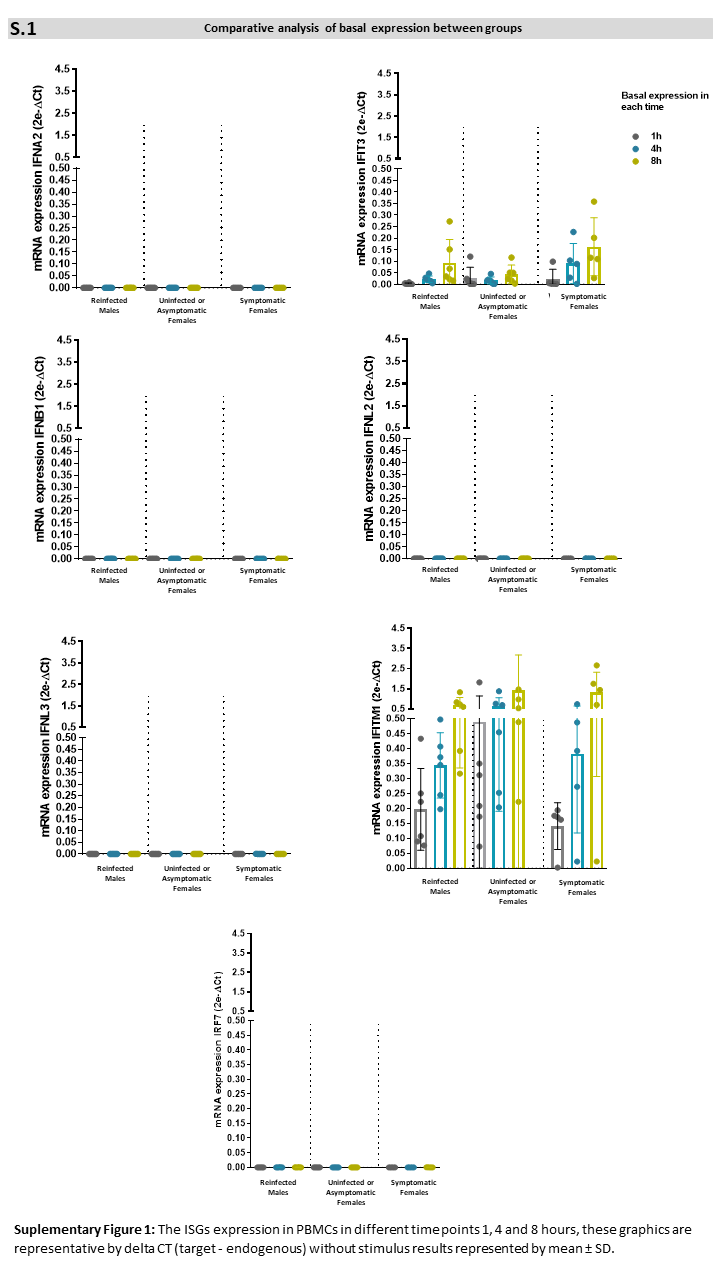

Supplement: Supplementary Figure 2 — The expression profiles of interferon-related genes (IFNA2, IFNB1, IRF7, IFIT3, IFITM1, IFNL2, and IFNL3) across specific immune cell types in COVID-19 patients, based on publicly available single-cell RNA sequencing (scRNA-seq) data. (A) Gene expression across various PBMC subtypes in blood samples from COVID-19 patients. Dot color represents gene expression levels, with a gradient from low (light yellow) to high (dark purple), while dot size indicates the percentage of cells within each population expressing each gene. (B) The expression levels of the same interferon-related genes in neutrophils from both normal (healthy) and COVID-19 conditions. Neutrophils, which are abundant in the airways of COVID-19 patients, play a key role in the antiviral immune response. The color gradient similarly represents gene expression intensity, and the dot size reflects the percentage of neutrophils expressing each gene in both conditions. [file Image1.tif]

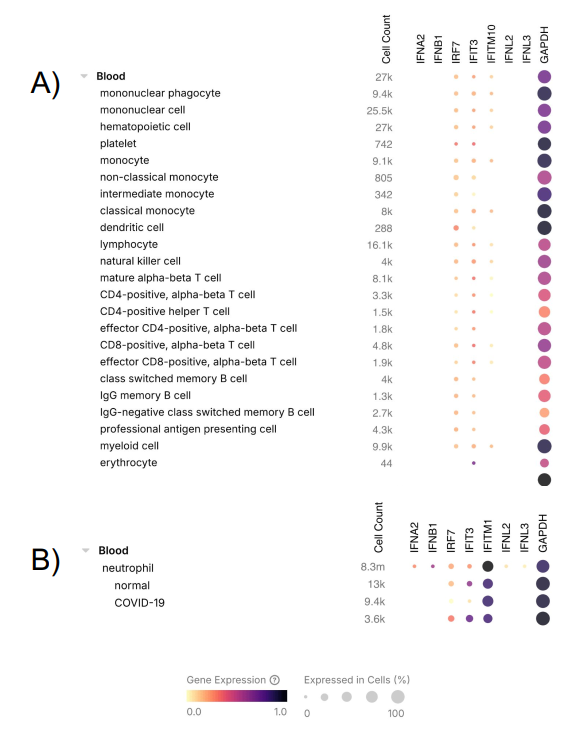

Supplement: Supplementary file 2 [file Image2.tif]
